# Seaweeds as Nutraceutical Elements and Drugs for Diabetes Mellitus: Future Perspectives

**DOI:** 10.3390/md22040168

**Published:** 2024-04-10

**Authors:** João Cotas, Silvia Lomartire, Leonel Pereira, Ana Valado, João Carlos Marques, Ana M. M. Gonçalves

**Affiliations:** 1 Marine Resources, Conservation and Technology, Marine Algae Lab, CFE—Centre for Functional Ecology: Science for People & Planet, Department of Life Sciences, University of Coimbra, 3000-456 Coimbra, Portugal; jcotas@uc.pt (J.C.); silvia.lomartire@student.uc.pt (S.L.); leonel.pereira@uc.pt (L.P.); 2 Polytechnic Institute of Coimbra, Coimbra Health School, Biomedical Laboratory Sciences, Rua 5 de Outubro—SM Bispo, Apartado 7006, 3046-854 Coimbra, Portugal; valado@estesc.ipc.pt; 3 Research Centre for Natural Resources, Environment and Society—CERNAS, Escola Superior Agrária de Coimbra Bencanta, 3045-601 Coimbra, Portugal; 4 MARE—Marine and Environmental Sciences Centre/ARNET-Aquatic Research Network, Department of Life Sciences, University of Coimbra, Calçada Martim de Freitas, 3000-456 Coimbra, Portugal; jcmimar@ci.uc.pt; 5 Department of Biology and CESAM, University of Aveiro, 3810-193 Aveiro, Portugal

**Keywords:** diabetes mellitus, high blood glucose levels, insulin, complications, marine natural products, multitargeted effects, antidiabetic agents, preclinical studies, peptides, polyphenols, polysaccharides, seaweeds, chemical diversity, mechanisms of action, therapeutic potential

## Abstract

Diabetes mellitus is a chronic metabolic condition marked by high blood glucose levels caused by inadequate insulin synthesis or poor insulin use. This condition affects millions of individuals worldwide and is linked to a variety of consequences, including cardiovascular disease, neuropathy, nephropathy, and retinopathy. Diabetes therapy now focuses on controlling blood glucose levels through lifestyle changes, oral medicines, and insulin injections. However, these therapies have limits and may not successfully prevent or treat diabetic problems. Several marine-derived chemicals have previously demonstrated promising findings as possible antidiabetic medicines in preclinical investigations. Peptides, polyphenols, and polysaccharides extracted from seaweeds, sponges, and other marine species are among them. As a result, marine natural products have the potential to be a rich source of innovative multitargeted medications for diabetes prevention and treatment, as well as associated complications. Future research should focus on the chemical variety of marine creatures as well as the mechanisms of action of marine-derived chemicals in order to find new antidiabetic medicines and maximize their therapeutic potential. Based on preclinical investigations, this review focuses on the next step for seaweed applications as potential multitargeted medicines for diabetes, highlighting the bioactivities of seaweeds in the prevention and treatment of this illness.

## 1. Introduction

Diabetes mellitus is a chronic metabolic disorder that has become a global health concern, affecting millions of people worldwide [1]. The disease is characterized by high blood glucose levels due to either insufficient insulin production or ineffective utilization of insulin [2]. Despite various treatment options available, the complications associated with diabetes, such as cardiovascular disease, neuropathy, nephropathy, and retinopathy, continue to be a major health burden [3].

In recent years, there has been a growing interest in marine natural products as a potential source of new drugs to treat diabetes and its associated complications [4]. Among the diverse range of marine organisms, seaweeds are of particular interest due to their unique chemical composition and medicinal properties [5]. Seaweeds produce a variety of bioactive compounds, including polysaccharides, polyphenols, and peptides, which have demonstrated antidiabetic effects in preclinical studies [6].

Polysaccharides isolated from seaweeds have been shown to improve insulin sensitivity, increase glucose uptake, and reduce blood glucose levels [7]. Additionally, polyphenols found in seaweeds have been shown to inhibit the activity of α-glucosidase, an enzyme that breaks down complex carbohydrates into glucose, leading to a reduction in postprandial blood glucose levels [8]. Peptides isolated from seaweeds have also demonstrated potential as antidiabetic agents, with some peptides showing insulin-like activity [9]. Despite these potential benefits of seaweeds compounds, there are scarce results from seaweed-based drugs for diabetes therapeutics.

Given the promising results of preclinical studies, seaweed-derived compounds have the potential to provide a rich source of new multitargeted drugs for the prevention and treatment of diabetes and its complications [10]. However, more research is needed to fully understand the mechanisms of action of these compounds and optimize their therapeutic potential. In this context, this review aims to provide an overview of the current research on seaweed-derived compounds and their potential as multitargeted drugs for the treatment of diabetes [11]. This review focuses on the seaweed compounds that are in preclinical and clinical studies and on the next road because of the various reviews based on seaweed compounds for diabetes therapeutic [6,9,12,13,14,15]. However, without exploiting the next steps for the drug-making industry or analyzing prevention methods to reduce the impact of diabetes on humankind, another recent mindset can be exploited, that is, the nutraceutical concept.

## 2. The Health Benefits of Seaweeds and Their Bioactive Compounds

**Figure 1:**
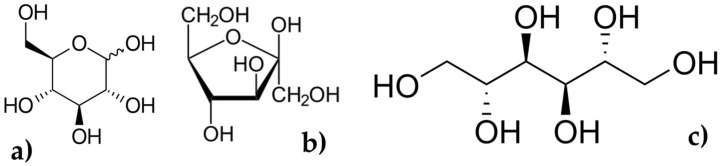
Sugars structures: (**a**) glucose; (**b**) fructose; (**c**) mannitol.

Seaweeds, or marine macroalgae, are aquatic plants that grow in marine environments. They are known for their diverse chemical composition and are an important source of nutrients, vitamins, and minerals [6,9,12,13,14,15]. Seaweeds contain various types of sugars, including glucose, fructose, and mannitol. These sugars (Figure 1) are important sources of energy for the human body and are metabolized through various pathways in the body [16].

Glucose is the primary sugar that is used by the body to produce energy. It is transported to the cells through the bloodstream, where it is taken up by the cells and converted to adenosine triphosphate (ATP), the molecule that provides energy for cellular processes [17]. 

Fructose is another sugar found in seaweeds, and it is metabolized in the liver. When consumed in excessive amounts, it can lead to insulin resistance, a condition in which the body’s cells become less responsive to insulin, leading to high blood sugar levels and an increased risk of developing type 2 diabetes [18].

Mannitol is a sugar alcohol that is commonly found in seaweeds. It is not metabolized by the body and is excreted unchanged in the urine. Mannitol has a mild laxative effect and is sometimes used as a sweetener in low-calorie foods [19].

**Figure 2:**
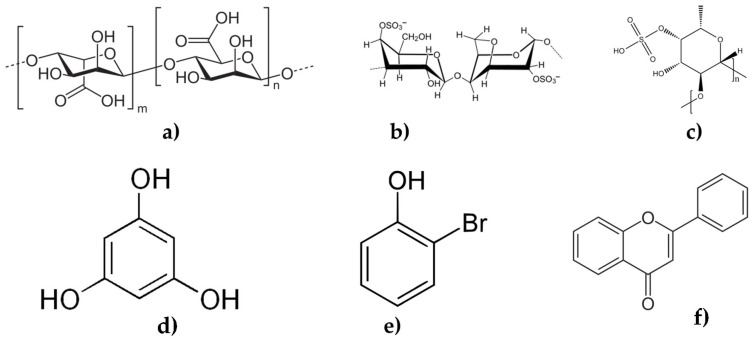
Seaweeds compound structure: (**a**) sodium alginate; (**b**) iota-carrageenan; (**c**) fucoidan; (**d**) phloroglucinol; (**e**) bromophenol; (**f**) flavonoid.

Seaweeds also contain dietary fibers, including alginate, carrageenan, fucoidan, and phenolic compounds (Figure 2). These fibers are not digested by the body and pass through the digestive tract relatively intact [20]. They help to promote feelings of fullness and satiety, which can aid in weight management. In addition to sugars and dietary fibers, seaweeds also contain a wide range of vitamins and minerals, including iodine, calcium, magnesium, iron, and vitamin C [14]. Iodine is particularly important for thyroid function, and a deficiency can lead to hypothyroidism, a condition in which the thyroid gland does not produce enough thyroid hormones [21].

The chemical composition of seaweeds makes them an important source of nutrients and dietary fibers that can benefit human health [15]. Among the sugars found in seaweeds, glucose and fructose are metabolized through various pathways in the body and can provide energy for cellular processes [16]. The dietary fibers in seaweeds can help to promote feelings of fullness and satiety, while the vitamins and minerals can help to support various bodily functions, including thyroid function [22].

Thus, we discuss a new mind shift to disease prevention using novel or more traditional foods, a concept known as nutraceuticals (where foods can prevent the appearance of diseases). Nutraceuticals’ concept is to focus on prevention; their position in human nutrition is one of the most significant fields of research, having far-reaching ramifications for consumers, healthcare professionals, regulators, food producers, and distributors. The definition of nutraceuticals and associated items varies according to the source. These goods can be classed according to their natural origins, pharmacological conditions, and chemical composition. Nutraceuticals are commonly classified as dietary supplements and functional foods.

### Seaweeds as Nutraceutical Ingredient

Global dietary studies have found that countries where seaweed is consumed on a regular basis have significantly less obesity and diet-related diseases, thus seaweed is considered a natural nutraceutical [23,24]. Early evidence dating back ten thousand years reveals that seaweeds were utilized as traditional food and complementary medicine [25]. Seaweeds have been consumed in various Asian countries, including China, Indonesia, the Philippines, South Korea, North Korea, Japan, and Malaysia, for centuries. However, the culinary use of seaweed originated primarily in Japan and China [26,27,28]. Recently, there has been a surge in the popularity of seaweed in Western countries, and seaweeds are now widely incorporated into the cuisine of the USA, South American nations, and European countries. 

This growing interest can be attributed to their functional properties and the introduction of Asian culinary traditions [25]. Seaweeds are considered low-calorie food options, while being abundant in vitamins, minerals, essential trace elements, polyunsaturated fatty acids, bioactive metabolites, proteins, polysaccharides, and dietary fibers. In addition to regular consumption, numerous studies have emphasized the health benefits of seaweed supplementation alongside a regular diet. For instance, pregnant Japanese women who regularly consume seaweed have experienced a reduction in depressive symptoms. Furthermore, seaweed consumption has been linked to a decreased risk of suicide in adults [23,29]. 

Seaweeds are also employed in the treatment and prevention of goiter, a condition caused by a deficiency of iodine in the diet [30]. Scientific investigations have highlighted various therapeutic effects of different types of seaweed against inflammation, obesity, diabetes, hypertension, and viral infections [31]. Clinical research has indicated that the regular consumption of *Undaria* seaweed can effectively reduce the risk of breast cancer in women [32]. Additionally, oral administration of seaweed extracts, such as *Fucus vesiculosus*, *Macrocystis pyrifera*, and *Saccharina japonica* (formerly *Laminaria japonica*) (Phaeophyceae), in combination with zinc, manganese, and vitamin B_6_, has shown promise in reducing osteoarthritis symptoms among a mixed population [33].

Seaweeds are recognized as a beneficial component of a nutritious diet, particularly in Eastern Asia [34]. Compared to Western countries, this region exhibits a lower prevalence of metabolic syndrome, which could be attributed to the consumption of fish, soy, and seaweeds [35,36,37,38]. A study analyzing the Korean National Health and Nutrition Examination Survey in 2005 revealed that the inclusion of algae in the diet was linked to a healthier dietary profile, characterized by higher proportions of legumes, fruit, fish, and dairy products. Additionally, after accounting for confounding factors, there was a notable trend suggesting a reduced progression of diabetes in males with a high intake of seaweeds [39].

**Figure 3:**
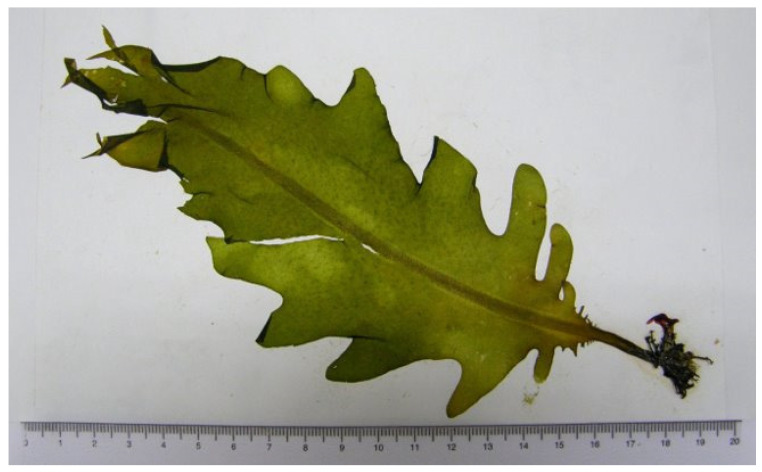
*Undaria pinnatifida* herbarium specimen.

Furthermore, the consumption of seaweeds was associated with a lower increase in the prevalence of metabolic syndrome among women [40]. When healthy volunteers consumed Mekabu, which refers to the sporophylls of *Undaria pinnatifida* (Figure 3), alongside a breakfast based on white rice, a decrease in postprandial glucose concentration was observed. This effect was attributed to the presence and viscosity of fucoxanthin, a compound found in Mekabu [41]. Furthermore, a recent study conducted in Korea involving over 4000 participants found an inverse relationship between insulin levels, insulin resistance, and the dietary intake of flavonols and flavones. This inverse association subsequently reduced the risk of type 2 diabetes mellitus [42].

Preclinical and clinical studies have provided evidence supporting the association between seaweed consumption and potential benefits for diabetes (Table 1). For instance, the consumption of crude fucoidan extracts derived from *U. pinnatifida* at a dosage of 45 mg/kg body weight was found to significantly prevent hyperglycemia in genetically diabetic (db/db) mice [43]. These mice serve as a genetic model for diabetes. This preventive effect was achieved through the improvement of glucose utilization and a decrease in fasting blood glucose and serum insulin concentrations. The study conducted by Kim et al. [44] in 2012 suggests a potential positive impact on glucose utilization.

Similarly, in another study using a mouse model of db/db mice, the administration of low-molecular-weight fucoidans (LMWF) at doses of 250 or 500 mg/kg resulted in a reduction in white adipose tissue weight, as well as decreased serum levels of total cholesterol, LDL cholesterol (LDL-C), and triglycerides. Jeong et al. [45] demonstrated that fucoidans activate AMP-activated protein kinase, which in turn improves glucose tolerance and insulin sensitivity. This improvement was associated with an increase in serum adiponectin concentrations. Additionally, the study revealed a decrease in intracellular lipid content, which was attributed to the enhancement of fatty acid oxidation.

Furthermore, in a separate study involving diabetic rats, fucoidan extracts obtained from the species *Saccharina japonica* were shown to stimulate the release of insulin from the pancreas, resulting in a reduction in plasma glucose concentrations. The study conducted by Wang et al. [46] highlights the potential of fucoidan extracts in managing diabetes. Overall, these in vivo studies provide supporting evidence for the potential benefits of seaweed consumption in relation to diabetes management.

A recent study by Murakami et al. [47] investigated the effects of *Gloiopeltis furcate* (Rhodophyta), an edible red seaweed, on diet-induced obesity in mice. The mice were subjected to a high-fat diet (HF) and treated with two doses of *G. furcata* for a duration of thirteen weeks. The findings revealed that the administration of *G. furcata* effectively mitigated the development of obesity, as evidenced by the suppression of weight gain and a reduction in white adipose tissue weight. Furthermore, the inclusion of *G. furcata* in the diet demonstrated a beneficial impact on obesity-associated metabolic disorders, including insulin resistance, hyperglycemia, hepatic steatosis, and dyslipidemia. Based on these results, it can be inferred that *G. furcata*, a traditional Japanese seaweed, holds potential as a beneficial ingredient for addressing metabolic disorders associated with obesity and diabetes.

*Sargassum horneri* (Phaeophyceae), also known as Akamoku in Japan, is a brown seaweed that naturally grows along the coast of East Asia [48]. Throughout Japan, Korea, and China, *S. horneri* has been utilized for centuries as both a food source and a traditional remedy for various disorders. Particularly in the Tohoku region of Japan, it has served as a local delicacy since ancient times. Recent studies conducted on animal models have garnered attention due to their findings that certain active constituents found in *S. horneri* have beneficial effects on health promotion and disease prevention [49,50]. *S. horneri* boasts high concentrations of polysaccharides, such as fucoidan and alginate. Fucoidan, a sulfated fucose-containing polysaccharide, has demonstrated a wide range of biological activities in animal and in vitro studies. These activities include anticancer, anticoagulant, immune-regulatory, anti-inflammatory, antiviral, antiobesity, and antidiabetic effects [51,52]. 

In a recent study, the potential of *S. horneri* to alleviate high-fat diet-induced obesity, diabetes, and hepatic steatosis in mice was examined. The researchers prepared freeze-dried, finely powdered *S. horneri* and incorporated it into a high-fat diet at weight ratios of 2% or 6% to feed the mice for a period of 13 weeks. This high-fat diet led to obesity, diabetes, hepatic steatosis, and hypercholesterolemia in the mice. However, supplementation with *S. horneri* effectively countered these effects by suppressing body weight gain, fat accumulation in adipose tissue and liver, and elevated serum glucose levels. Additionally, *S. horneri* improved insulin resistance. Analysis of fecal samples indicated that *S. horneri* promoted the excretion of triglycerides and increased fecal polysaccharide content. Moreover, extracts from *S. horneri* displayed inhibitory effects on pancreatic lipase activity in vitro. These results collectively demonstrate that *S. horneri* can ameliorate metabolic diseases induced by a high-fat diet, possibly through the suppression of intestinal fat absorption [53]. *S. horneri* is also distinguished by its elevated levels of bioactive polysaccharides and fucoxanthin in comparison to popular edible brown seaweeds like Wakame (*Undaria pinnatifida*) seaweed and Japanese tangle (*Saccharina japonica*), which is a subject of considerable health interest. The compound fucoxanthin, found in *S. horneri*, showcases diverse benefits, including antiobesity and antidiabetic properties, as substantiated by sixteen-week, double-blind, randomized, placebo-controlled studies, highlighting its efficacy in treating human obesity. Notably, both fucoxanthin and its derivative fucoxanthinol exhibit the ability to decrease lipase activity. The antiobesity impact of *S. horneri* is attributed to the hindrance of pancreatic lipase, leading to a decrease in intestinal lipid absorption and subsequent accumulation in adipose tissue and the liver. Moreover, the anti-inflammatory attributes of *S. horneri* may contribute to addressing obesity and diabetes, with its major components—fucoidan, alginate, and fucoxanthin—taking the forefront in delivering these beneficial effects [53].

**Table 1:** Investigations of antidiabetic properties of seaweeds bioactive compounds.

Seaweed	Bioactive Compound	Dosage Used in Preclinical/Clinical Study	Bioactivity Detected	Reference
*Undaria pinnatifida*	Fucoidan	Preclinical study: fucoidans were administered by oral gavages in 45 mg/kg, 7 days/week for 6 weeks, in genetically diabetic mice	Prevent hyperglycemia in genetically diabetic mice	[44]
*Undaria pinnatifida*	Fucoidan	Preclinical study: mice were treated orally for 6 weeks with LMWF (250 or 500 mg/kg)	Reduction in white adipose tissue weight in mice, decreased serum levels of total cholesterol, LDL-C, and triglycerides	[45]
*Saccharina japonica*	Fucoidan	Preclinical study: Wistar diabetic rats were treated for 18 days with FPS (100, 300, and 600 mg/kg body weight)	The stimulation of the release of insulin from the pancreas in mice led to reduction in the blood glucose level, which was significantly reduced when compared with the diabetic control group	[46]
*Gloiopeltis furcata*	Crude extract	Preclinical study: mice were fed a high-fat diet (60% energy as fat) supplemented with 2% (*w/w*) or 6% powdered *G. furcata* for 13 weeks	Suppression of weight gain and a reduction in white adipose tissue weight in mice	[47]
*Sargassum horneri*	Crude extract	Preclinical study: mice fed a high-fat diet supplemented with 2% (*w/w*) or 6% powdered *S. horneri* for 13 weeks	Alleviate high-fat diet-induced obesity, diabetes, and hepatic steatosis in mice	[53]
*Undaria pinnatifida*	Whole algae	Clinical study: 12 healthy subjects consumed 70 g of Wakame per 3 days	Decrease in postprandial glucose concentration in patients	[41]

However, it is important to note that not all these studies provided a comprehensive understanding of the factors responsible for the beneficial properties of seaweed consumption in diabetes management. As a result, further investigation is warranted to explore the potential of various beneficial components in seaweed and their potential mechanisms of action in preventing the development of type 2 diabetes mellitus.

Thus, using seaweeds as nutraceuticals can be a disease prevention technique and welfare system that can be applied in human traditional foods; however, when diabetes appears in humans, there is need for therapeutics, and novel natural drugs can be a key for diabetes therapeutics without the actual cons of the actual approved drugs.

## 3. Seaweed Compounds as Potential Diabetes Therapeutics

**Figure 4:**
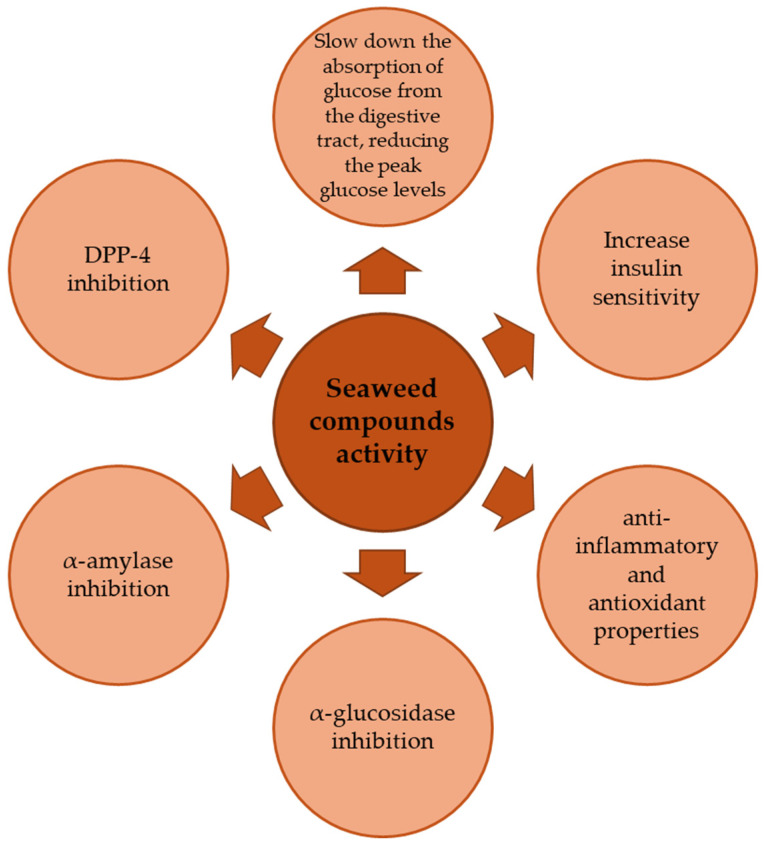
Mechanisms of action involved in antidiabetic activities of seaweed bioactive compounds.

When diabetes disease appears in humans, there is a need to initiate therapy to control and ameliorate the diabetes-associated problems. The seaweeds are one of the potential natural sources from which to find new and advanced therapy to treat and reduce diabetes-related problems, working mostly as glycemic modulators and showing insulin-like activity (Figure 4) [6,9,12,13,14,15]. 

Furthermore, this theme is very targeted in various studies and reviews (Table 2) [6,9,34,38,39,54,55,56,57,58,59,60,61,62,63,64].

Seaweeds contain a variety of non-nutritive bioactive compounds, including fucoxanthin, phlorotannins, and sulfated polysaccharides, known for having antidiabetic properties without causing side effects. These compounds primarily function by inhibiting carbohydrate-hydrolyzing enzymes, such as α-amylase and α-glucosidase, thereby regulating the postprandial increase in blood sugar levels and insulin resistance. However, there is considerable variation in seaweed extract preparation protocols, including the type of solvent and conditions used, making it challenging to compare the optimal enzymatic inhibition of carbohydrate hydrolysis. Supplementation of seaweed extracts and their bioactive compounds enhances glucose uptake in muscle cells by regulating AMPK and Akt pathways. Additionally, the downregulation of pro-inflammatory cytokine mRNA expression and the upregulation of adiponectin levels contribute to their beneficial effects. Seaweeds also improve lipid--lipoprotein fraction levels, mitigating diabetes-associated comorbidities and dyslipidemia. Recent research highlights the positive effects of seaweed bioactive compounds on improving the composition of gut microbiota due to their bioavailability [65]. Bioavailability involves two critical stages: bioaccessibility and bioactivity. Bioaccessibility pertains to the release of a consumed component from its food matrix, making it accessible for absorption in the colon [66]. Bioactivity, on the other hand, encompasses the transportation of a medicinal or food component to the target tissue, interaction with other biomolecules, biotransformation/metabolism, and the induction of a physiological response [20,67]. The interplay between bioavailability, the human digestive system, and the food matrix is crucial. Understanding the kinetics of seaweed in relation to stability is essential for gaining insights into its impact on the digestive process, ensuring improved food safety, and maintaining a consistent product with stable chemicals that are bioavailable to humans, thus promoting human welfare.

Despite the importance of these considerations, there is a noticeable lack of bioavailability studies specifically focused on seaweeds. Most existing studies tend to concentrate on isolated compounds, particularly seaweed polysaccharides. Raw seaweed biomass contains proteins, polysaccharides, lipids, and minerals, but their variable intrinsic composition may impede their biological functionality in the human body. While laboratory-based mimics/models attempt to replicate conditions in the digestive system, in vivo investigations aim to provide precise descriptions of the digestion and fermentation pathways of seaweed isolated proteins, polysaccharides, and lipids by gut microbiota [68,69].

**Table 2:** Physiological effects of seaweed compounds investigated for the treatment of diabetic diseases.

Detected Activity	Seaweed Compound	Physiological Effects of Seaweed Compounds	References
Anti-inflammatory properties	Polysaccharides Polyphenols	Reduced inflammatory effect in chronic inflammation associated with insulin resistance	[7,70,71,72]
Antioxidant properties	Polysaccharides	Antioxidants protect against the development of diabetic complications	[60,73]
Change in lipid metabolism	Polysaccharides Polyphenols	Reduced levels of triglycerides and LDL-C	[60,62,74,75,76]
α-glucosidase inhibition	Polysaccharides Polyphenols Peptides	Slowed down the absorption of glucose, reducing the peak glucose levels in blood	[7,10,70,77,78,79,80,81,82,83]
α-amylase inhibition	Polysaccharides	Slower digestion and absorption of carbohydrates to promote better glycemic control	[8,84]
DPP-4 inhibition	Polysaccharides Peptides	Inhibition of DPP-4, which inactivates hormones involved in insulin secretion	[85,86,87,88,89,90]

### 3.1. Polysaccharides as Glycemic Modulators 

Seaweed polysaccharides are complex carbohydrates that are abundant in seaweeds. These polysaccharides have been shown to have a variety of potential health benefits, including the ability to regulate blood sugar levels and treat diabetes. Studies have also shown that seaweed polysaccharides may have additional benefits for individuals with diabetes. For example, they have been found to have anti-inflammatory effects, which is important because chronic inflammation is associated with insulin resistance, a key feature of type 2 diabetes [7,70]. Seaweed polysaccharides have also been shown to have antioxidant properties. This is significant because people with diabetes are more prone to oxidative stress, which can lead to damage to cells and tissues. Antioxidants can help to counteract this damage and protect against the development of diabetic complications [60,73].

Furthermore, seaweed polysaccharides have been shown to have beneficial effects on lipid metabolism. In animal studies, they have been found to reduce levels of triglycerides and LDL-C, which are risk factors for cardiovascular disease, a common complication of diabetes [62,74,75]. While seaweed polysaccharides show promise as potential treatments for diabetes, it is important to note that further research is needed to determine their safety and effectiveness in humans. Additionally, the optimal dosage and mode of administration (e.g., as a supplement or incorporated into food) also need to be established [77,91].

Several investigations confirmed that seaweed polysaccharides, such as fucoidan, laminarin, and alginate, can modulate glycemic response in the body. They do this by slowing down the absorption of glucose from the digestive tract, thereby reducing the peak glucose levels that occur after a meal. This can help to prevent sudden spikes in blood sugar levels, which are common in people with diabetes [70,78].

Fucoidan, for example, has been shown to increase insulin sensitivity in animal studies, which can help the body to use insulin more effectively and maintain healthy blood sugar levels. It has also been shown to have anti-inflammatory and antioxidant properties, which can further support the management of diabetes [79,80].

Laminarin, another seaweed polysaccharide, has been shown to inhibit the activity of α-glucosidase, an enzyme that breaks down carbohydrates into glucose. By inhibiting this enzyme, laminarin can reduce the amount of glucose that is absorbed from the digestive tract and therefore lower blood sugar levels [10,81].

Alginate, on the other hand, forms a gel-like substance in the digestive tract, which can slow down the digestion and absorption of carbohydrates. This can help to prevent sudden spikes in blood sugar levels and promote better glycemic control [8,84].

Nevertheless, seaweed polysaccharides represent an exciting avenue for the development of new treatments for diabetes and related conditions. As research in this field continues to advance, it is hoped that these natural compounds will become an important tool in the fight against this growing health epidemic [22,92].

Overall, the ability of seaweed polysaccharides to modulate glycemic response and improve insulin sensitivity makes them promising candidates for the development of new treatments for diabetes. However, more research is needed to fully understand their mechanisms of action and potential therapeutic applications in humans [13,93].

### 3.2. Phenolic Compounds as α-Glucosidase Inhibitors

One of the ways the body regulates blood sugar levels is through the action of an enzyme called α-glucosidase, which breaks down complex carbohydrates into simple sugars that can be absorbed by the body [94]. α-glucosidase inhibitors are a type of medication used to treat diabetes that work by blocking the action of this enzyme, thereby reducing the amount of sugar that is absorbed into the body [95].

Polyphenols are compounds found in many plants and seaweeds, which possess antioxidant and anti-inflammatory properties [71,72]. Studies have shown that some terrestrial plants have polysaccharides that express antidiabetic properties.

Mangroves possess unique biochemical functions in their ecosystem and are considered as a source of novel natural/biological products. Mangroves are rich resources of compounds like polyphenols and tannins. Further, mangrove leaves also possess phenolic compounds, alkaloids, and flavonoids, which serve as novel bioactive compounds. Traditionally, mangrove plants are used in folklore medicine for the treatment of several ailments including diabetes throughout the world. Many plants are a rich source of potent antidiabetic drugs, and these herbal preparations are devoid of any side effects. Approximately 400 plants and their secondary metabolites, namely, alkaloids, carotenoids, flavonoids, glycosides, polyphenolics, terpenoids, and tannin molecules, have been used for treating diabetes [55]. The research delved into the antidiabetic properties of five specific wild plants found in Bangladesh. The findings indicate that these plants, examined in the study, hold promise as rich sources of essential nutrients and could be utilized as ingredients for crafting functional foods. The samples demonstrated substantial mineral composition, vitamin C content, and total phenolic and total flavonoid levels, along with noteworthy antioxidant capacities as assessed by DPPH, FRAP, and TEAC assays. Additionally, the study provides preliminary evidence supporting the efficacy of the selected wild plants in inhibiting the α-amylase enzyme. Consequently, incorporating these wild plant species into one’s diet may offer health benefits, owing to their potential to inhibit α-amylase enzyme activity and their elevated antioxidant and nutritional values [96].

Some seaweed-derived polyphenols have been shown to have α-glucosidase inhibitory properties, meaning they can block the action of the enzyme and reduce the amount of sugar that is absorbed into the body [7,97].

The potential of marine algal polyphenols as α-glucosidase inhibitors for the treatment of diabetes has been investigated [9,95]. These compounds have been shown to have α-glucosidase inhibitory activity comparable to or even superior to that of conventional drugs used to treat diabetes. In addition, seaweed polyphenols may also have other health benefits, such as lowering cholesterol and preventing cardiovascular disease [60,76].

In summary, marine algae polyphenols are a promising group of natural compounds that may have therapeutic potential for the treatment of diabetes, as they have been shown to have α-glucosidase inhibitory activity comparable to or superior to that of conventional drugs used to treat disease. In addition, these compounds may also have other health benefits, making them an interesting option for the development of new treatments for diabetes and other related metabolic diseases [6,98].

Therefore, as both terrestrial and marine polyphenols showed potential for antidiabetic activity, it is more likely that patients affected with diabetic diseases can obtain natural treatment based on natural formulas.

### 3.3. Peptides with Insulin-like Activity

Seaweed peptides have recently emerged as a promising avenue for the treatment of diabetes [99]. Traditional treatments for diabetes include insulin injections and oral medications, but these can have side effects and are not always effective. Seaweed is a rich source of bioactive peptides, which are short chains of amino acids that have a range of biological activities [60]. Recent studies have shown that certain seaweed peptides have insulin-like activity and could potentially be used to treat diabetes [7,82]. In vitro studies have demonstrated the ability of seaweed peptides to stimulate glucose uptake and improve insulin sensitivity [83]. In animal studies, oral administration of seaweed peptides was shown to improve glucose tolerance and insulin sensitivity in obese mice and reduce blood glucose levels in diabetic rats [77].

Dipeptidylpeptidase-4 (DPP-4) is a serine protease responsible for regulating specific circulating peptide hormones by selectively cleaving 2N-terminal amino acids, namely, Xaa-Pro and Xaa-Ala. Its role includes the inactivation of two incretin hormones, GLP-1 and glucose-dependent insulinotropic peptide, both of which enhance insulin secretion. Consequently, inhibiting DPP-4 is a crucial molecular target in the treatment of diabetes. Therefore, it is essential for managing type 2 diabetes mellitus to identify DPP-4 inhibitor peptides from natural sources and subsequently develop biofunctional foods, reducing reliance on synthetic inhibitors. The potential of *Ulva* spp. (Chlorophyta) protein hydrolysate to inhibit DPP-4 was assessed, revealing that a peptide fraction containing a DPP-4 inhibitor was isolated from *Ulva* spp. hydrolysate through a fractionation and purification process. The identified peptide, SLAVSVH, demonstrated inhibitory activity against the DDP-4 enzyme. The presence of hydrophobic and branched amino acids in SLAVSVH aligns with its inhibitory properties, making it a promising natural peptide inhibitor. Consequently, SLAVSVH could serve as a valuable biofunctional ingredient derived from *Ulva*, contributing to the development of functional foods [85]. 

Significantly, both the crude hydrolysates derived from *Palmaria palmata* (Rhodophyta) and the isolated peptides obtained from this source have exhibited the capability to inhibit DPP-4 in vitro [86,87]. Additionally, a study revealed that the twice-daily chronic administration of a crude *P. palmata* protein hydrolysate (PPPH) and Alcalase/Flavourzyme PPPH led to improved glycemic control in streptozotocin-induced diabetic mice [88]. Another research study investigated the metabolic advantages of protein hydrolysates derived from the macroalgae *P. palmata*, which had previously demonstrated inhibitory effects on DPP-4 activity in vitro [89]. To assess the acute in vivo effects of Alcalase/Flavourzyme PPPH administration on glucose tolerance and satiety, the study utilized overnight-fasted mice. The results indicated that Alcalase/Flavourzyme PPPH positively impacted glycemia and insulin production in streptozotocin-induced diabetic mice, displaying insulinotropic effects and enhancing glucose tolerance. Furthermore, PPPHs were found to directly influence the incretin effect by upregulating GLP-1 and GIP, thereby stimulating insulin secretion from pancreatic β cells. 

The isolation and structural characterization of a sulfated galactofucan from the brown seaweed *Padina tetrastromatica* (Paheophyceae) has been assessed for its antihyperglycemic activities using different in vitro models. The purified polysaccharide was analyzed for its potential to attenuate the proteolytic enzyme DPP-4 and carbolytic enzymes (*α*-amylase and *α*-glucosidase). In vitro studies on DPP-4 and carbolytic enzymes (IC_50_ less than 1 mg mL^−1^) with the sulfated galactofucan showed its promising attenuation potential against these enzymes with respect to normal control. *P. tetrastromatica* was demonstrated to be a valuable natural resource of bioactive sulfated galactofucan exhibiting potential activities against the proteolytic DPP-4; therefore, the isolated sulfated galactofucan could be developed as a novel bioactive agent attenuating the hyperglycemia-related disorders [90].

While the potential of seaweed peptides as a natural and safe alternative to traditional diabetes treatments is promising, more research is needed to determine their safety and efficacy in humans. Nonetheless, the ability of seaweed peptides to mimic the action of insulin and regulate blood sugar levels represents a significant opportunity for the development of new treatments for diabetes [11,100]. Other studies have investigated the mechanisms by which seaweed peptides exert their insulin-like effects. One study found that a seaweed peptide activated an insulin receptor signaling pathway in muscle cells, like the action of insulin itself. This suggests that seaweed peptides may work by directly mimicking the action of insulin in the body [101,102].

While seaweed peptides show promise as diabetes treatments, there are still many questions that need to be answered. For example, it is not yet clear which specific seaweed peptides are most effective or whether they are safe for long-term use. Clinical trials in humans will be necessary to fully evaluate the safety and efficacy of seaweed peptides as diabetes treatments [61,103]. Nonetheless, the potential of seaweed peptides as a natural and safe alternative to traditional diabetes treatments is exciting. Seaweed is a sustainable and widely available resource that could provide a new source of therapeutic compounds for diabetes and other metabolic disorders [83,104].

### 3.4. Mechanisms of Action of Seaweed Compounds in Glycemic Regulation and Prevention of Diabetic Complications

Currently, the mechanism responsible for the antidiabetic activity of seaweed extracts and compounds remains unknown, but it is likely attributed to antioxidant enzymes that combat free radicals, thereby alleviating hyperglycemia induced by oxidative stress and associated hyperlipidemia. In vitro, seaweed-based extracts and compounds demonstrate the ability to inhibit carbohydrate-hydrolyzing enzymes, and in vivo, they reduce blood glucose levels in both random and postprandial blood glucose testing.

In certain animal studies, these extracts and compounds have shown promise in reducing weight gain, potentially by downregulating the mRNA expression of pro-inflammatory cytokines and upregulating anti-inflammatory cytokines. Notably, a seaweed-based antidiabetic medicine was developed and commercially introduced as Cadalmin^TM^ Antidiabetic Extract for treating type 2 diabetes. Despite these advancements, further extensive research in this field is essential [105].

**Figure 5:**
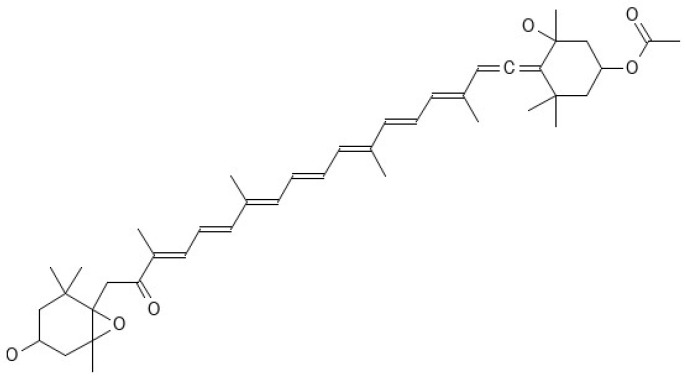
Fucoxanthin structure [76,106,107].

Seaweed compounds have been shown to have several mechanisms of action that can help to regulate glycemic levels and prevent diabetic complications [6,13]. Overall, the mechanisms of action of seaweed compounds in glycemic regulation and prevention of diabetic complications are diverse and complex. However, the evidence suggests that seaweed compounds have great potential as natural and safe alternatives to traditional diabetes treatments [84,89,90]. For example, inhibiting α-glucosidase can help slow the absorption of carbohydrates and prevent spikes in blood glucose levels, since α-glucosidase is an enzyme that breaks down complex carbohydrates into simple sugars. Seaweed compounds such as fucoxanthin (Figure 5) have been shown to inhibit α-glucosidase activity [76,106,107].

Moreover, the gut microbiota plays an important role in glycemic regulation and metabolism. Seaweed compounds such as fucoidan and laminarin have been shown to modulate the gut microbiota, promoting the growth of beneficial bacteria and reducing inflammation [108,109].

Seaweed peptides have been shown to have insulin-like activity, which means they can stimulate glucose uptake and improve insulin sensitivity. This can help to regulate blood glucose levels and prevent hyperglycemia, a common complication of diabetes [64,70].

Diabetes is associated with oxidative stress, which can lead to tissue damage and complications such as diabetic neuropathy and retinopathy. Seaweed compounds such as phlorotannins have been shown to have potent antioxidant activity, which can help to prevent or reduce oxidative stress in the body [60,110,111].

Inflammation is another common complication of diabetes and can contribute to the development of cardiovascular disease, neuropathy, and other complications. Seaweed compounds such as fucoidan and fucoxanthin have been shown to have anti-inflammatory activity, which can help to reduce inflammation and prevent complications [112,113,114].

Seaweeds include an elevated yield of bioactive substances such as polysaccharides, peptides, and polyphenols, which have been examined for their possible health benefits, including antidiabetes characteristics as demonstrated above. Further research is needed to fully understand the mechanisms of action of these compounds and optimize their therapeutic potential [13,70]. While these assays indicate promise, additional studies are needed to completely understand the mechanisms of action as well as to discover the ideal dosage and long-term benefits of seaweed components in the treatment of diabetes. 

The seaweed potential is evident in other reviews [6,9,12,13,14,15]. However, there is still a long journey for a seaweed compound to reach the commercial level and be an available drug for diabetes therapy.

## 4. Preclinical and Clinical Studies on the Efficacy and Safety of Seaweed Compounds for the Treatment of Diabetes

Over the past four decades, there has been a significant expansion in the intricacies of drug development. This expansion necessitates a preclinical phase in drug research, the submission of an investigational new drug (IND) application, and extensive clinical testing before obtaining marketing clearance from medicament/drug agencies. Generally, both biologics license applications (BLAs) and new drug applications (NDAs) undergo thorough review processes before receiving approval. After approval, the performance of the drug is subject to further scrutiny through postmarketing investigations by regulatory agencies. 

The aim is to promptly deliver safer and more effective therapies to patients following a comprehensive medical examination. The drug development process of the U.S. Food and Drug Administration (FDA) and the European Medicines Agency (EMA) comprises four crucial components, each with multiple phases and stages. The stages of drug development are as follows: (1) Discovery and development: identifying compounds crucial in addressing illnesses. (2) Preclinical studies: assessing the efficacy, toxicity, and pharmacokinetic data of the new drug in nonhuman subjects through in vivo, in vitro, and ex vivo tests on living creatures, cells, animals, or nonliving organisms and tissue extracts. (3) Clinical drug development: involving volunteer studies and clinical trials to adapt the medication for human consumption. This step comprises three phases: Phase I (<100 patients), Phase II (100–500 patients), and Phase III (1000–5000 patients). (4) National and international medicament/drug agency approval review: after obtaining approval and becoming commercially available, national and international agencies conduct postmarket safety monitoring [115].

For seaweeds to be classified as drug, there is a need to conduct preclinical and clinical studies to understand the compound safety (compound chemical characterization), biochemical safety (compound reaction and stability), and pharmacological activities (lethal dosage, IC_50_, and cytotoxicity), and if every step is good and approved, the compound can be further analyzed in pharmacodynamic and pharmacokinetics studies. Before it can be tested in in vivo assays in humans, it is necessary to have all the data approved by drug agencies to be used as therapeutic.

Several preclinical and clinical studies have been conducted to investigate the efficacy and safety of seaweed compounds for the treatment of diabetes. Here are some key findings from these studies [9,10,92]: most of the seaweed compounds are still in preclinical studies, and there is a reduced compound list for clinical trials due to the wide range of methodology; however, high number of them are not support by drug agencies so they are only preclinical studies without being certified by drug agencies. Numerous preclinical studies have demonstrated the potential of seaweed compounds for the treatment of diabetes. For example, studies in animal models of diabetes have shown that fucoidan can improve glucose tolerance and insulin sensitivity, reduce blood glucose levels, and protect against diabetic nephropathy [92,116]. Other studies have demonstrated the efficacy of laminarin and phlorotannins in improving glycemic control and reducing oxidative stress and inflammation [10,117].

Several clinical studies have been conducted to investigate the efficacy and safety of seaweed compounds in humans with diabetes. For example, a randomized controlled trial involving 60 patients with type 2 diabetes found that supplementation with fucoidan for 12 weeks improved glycemic control, reduced insulin resistance, and lowered levels of inflammatory markers [13,118]. Another study involving 29 patients with type 2 diabetes found that daily consumption of a seaweed-derived dietary supplement for 12 weeks improved glycemic control and reduced oxidative stress [119].

Clinical studies have generally found that seaweed compounds are safe and well tolerated when consumed in moderate amounts [120]. However, some compounds such as fucoidan can have anticoagulant effects, so caution should be exercised in patients taking blood-thinning medications [121]. Additionally, some seaweed species can contain high levels of iodine, which can cause thyroid dysfunction in individuals with iodine sensitivity or deficiency [122]. Despite the promising results of preclinical and clinical studies, there are some limitations to the current research. Many of the studies have been small in scale and short in duration, and there is a lack of standardization in the preparation and dosing of seaweed compounds [123]. Further research is needed to confirm the efficacy and safety of these compounds in larger and longer-term studies and to determine optimal dosing and preparation methods [91].

In summary, preclinical and clinical studies have shown that seaweed compounds have great potential for the treatment of diabetes. While further research is needed to confirm their efficacy and safety, these compounds represent a promising area of investigation for the development of natural and safe diabetes treatments [65].

## 5. Potential for the Development of New Multitarget Drugs from Seaweed Compounds

Seaweed compounds have been used for centuries in traditional medicine for the treatment of various ailments, and modern research has revealed their potential as nutraceuticals [102,124]. However, the new road to finding natural multitarget drugs, which are designed to target multiple pathways or proteins involved in a disease process, leads to an increasing interest in seaweed compounds. This approach can lead to more effective therapies with fewer side effects, as it allows for a more comprehensive and targeted approach to treatment [125,126].

Seaweed compounds have shown promise in this regard, as many of them have been found to have activity against multiple targets. For example, fucoidan, a sulfated polysaccharide found in brown seaweeds, has been shown to have anti-inflammatory, anticancer, and antiviral properties, as well as potential for the treatment of diabetes and cardiovascular disease [78,127].

Other seaweed compounds, such as phlorotannins and bromophenols, have also been found to have multitarget activity. Phlorotannins, which are found in brown seaweeds, have been shown to have anti-inflammatory, anticancer, antiobesity, and antidiabetic properties [128,129]. Bromophenols, which are found in red and brown seaweeds, have been found to have anti-inflammatory, antiviral, and anticancer properties [63,128,130].

The development of new multitarget drugs from seaweed compounds is an active area of research, and there is a growing interest in the potential of these compounds for the treatment of a wide range of diseases. However, further research is needed to fully understand their mechanisms of action and potential side effects, as well as to optimize their pharmacokinetic and pharmacodynamic properties for clinical use [11,128,131]. 

The seaweed sector for human consumption, as a novel food product, faces challenges such as the absence of standardized quality and safety processes and regulatory guidelines. Analytical techniques are now available to assess the nutritional value of seaweeds, offering improved quality and safety checks for seaweed-based products. However, there is a pressing need for advancements in standardizing methodologies and findings. The desire to collaborate with the scientific community to establish applicable rules and laws for seaweed farmers and the industry is growing across the Asian, European, and American continents [132].

Accurate identification of algae metabolites holds paramount importance in fully unlocking the chemical potential of these natural matrices using fingerprinting approaches. To address this gap, metabolomics emerges as a potent tool for obtaining a comprehensive metabolic profile of minimally explored heterogeneous seaweed extracts, facilitating the identification and quantification of their metabolites. Leveraging the analytical power and flexibility of metabolomic approaches, several researchers have gained detailed insights into the biosynthesis of amino acids, phenolic compounds, phytosterols, terpenoids, lipids, carotenoids, and pigments in a diverse range of seaweeds and microalgae. Furthermore, the integration of metabolomics with other high-throughput technologies such as genomics and proteomics opens extensive possibilities in the field of biotechnology to investigate numerous applications in the realms of food, nutraceuticals, and pharmaceuticals [57].

## 6. Challenges and Opportunities in the Research and Development of Drugs from Seaweeds for the Treatment of Diabetes

The research and development of nutraceutical and drugs from seaweeds for the treatment of diabetes present both challenges and opportunities. The most important challenges are in the identification and isolation of the active compounds because seaweeds contain a complex mixture of bioactive compounds, and the identification of the specific compounds responsible for the antidiabetic activity can be challenging [133]. Thus, to be considered a drug, the compound needs to be isolated and characterized; if not, it can only be considered traditional/herbal medicine or, at most, a nutraceutical solution. This theme develops into another question regarding the standardization of the extracts. The extraction process of the bioactive compounds from seaweeds can vary depending on the species, location, and extraction method used, leading to inconsistent results. Standardization of the extracts is essential for the reproducibility of the antidiabetic activity [134,135]; without the standardization of the extraction process and control safety measures, it will be very difficult to comply with international agencies for drugs or even for food safety. Due to the diverse metabolism and toxicity exhibited by various bioactive compounds in biological systems, it is imperative to investigate them for dose optimization and explore their potential therapeutic and pharmaceutical applications. Consequently, a greater emphasis on conducting additional clinical trials and bioavailability studies focusing on whole seaweeds is necessary to obtain a comprehensive evaluation of their nutraceutical properties [65].

The seaweed sector faces several challenges that warrant consideration. In terms of human health, it is crucial to acknowledge certain drawbacks associated with the use and treatment of algae in culinary practices. Seaweeds possess unique biosorption characteristics due to their cell wall structure, allowing them to absorb minerals and trace elements from their surroundings. While this accumulation capability is beneficial, it also leads to the potential accumulation of hazardous materials, including cadmium, lead, mercury, inorganic arsenic, and excessive iodine. Unregulated distribution of seaweed rich in these components poses health risks to consumers, with potential consequences such as thyroid dysfunction from excessive iodine intake and toxicity from metals like mercury, lead, cadmium, and inorganic arsenic. Variability in seaweed species, coupled with external factors such as geographic location and processing methods, influences the composition of these elements. Concerns among food regulators about the potential toxicological profile of seaweed underscore the importance of monitoring and regulating its consumption for public health and safety [115,136,137]. 

Chemical intoxication poses a range of health risks, impacting neurological, circulatory, enzymatic, endocrine, and immunological systems. It is crucial to monitor and restrict the consumption of seaweeds to mitigate potential harm from excessive exposure to certain substances [54,138]. The European Commission’s recommendation (2018/464) advises restricting the consumption of certain European macroalgae species, underscoring the necessity for a thorough characterization of macroalgae intended for consumption [139]. According to a report by the FAO, there is currently no universally accepted global regulation for the utilization of seaweed and seaweed-based products in food applications. Nevertheless, some countries have implemented regulations addressing food safety hazards related to seaweed. The European Union has tasked CEN/TC 454 with standardizing algae and algal products, encompassing the development of standards in areas lacking seaweed analysis methods (such as species identification, pigments, sugars, proteins, and lipids). Recently, the FDA officially categorized unprocessed seaweed as a raw agricultural commodity (RAC) in the United States, subjecting it to the general regulations of the Federal Food, Drug, and Cosmetic Act. France has also imposed strict limits on inorganic arsenic, cadmium, lead, and mercury in edible seaweeds, and China has set regulatory limits for cadmium. Consumer-level food safety risks associated with seaweed have prompted advisory notices in Japan, Ireland, and Norway. There is an urgent need for globally standardized regulations governing the use of seaweed as food [105].

Regarding contaminants, seaweed tends to accumulate metals and iodine from its surrounding waters. Consequently, a careful assessment of algae composition and potential food safety risks, including the presence of microorganisms and elevated levels of certain elements, is vital. Generalizations about food safety risks should not be applied universally to entire groups of seaweed, and references for different species must be categorized for each component and each site. Several studies highlight the benefits of seaweed aquaculture development, emphasizing its simplicity, eco-friendly characteristics, low initial investment (in comparison to animal aquaculture), and role in cleaning coastal areas to enhance biodiversity and fisheries [139].

However, seaweeds are aquatic organisms and even in aquaculture, they can be difficult to maintain so that all the compounds are instable. There is a need to observe the targeted compound(s) for biochemical similarity between batches. Thus, the safety of the extracts and compounds derived from seaweeds is crucial for their use as drugs. Toxicological studies are required to determine their safety profiles [140,141].

The next phase in seaweed aquaculture involves the implementation of an information-driven cultivation system. This system enables more precise control over cultivation and the prediction of seaweed nutritional values and bioactive compound metabolization through a specific kinetic model. The goal is to achieve consistent values across different batches of seaweed cultivated in the same area. The focus is on enhancing seaweed safety postcultivation by addressing concerns such as water nutrient fluctuations and potential contaminations. Real-time sensors providing critical data on offshore system parameters like nutrients, pH, temperature, and salinity can be integrated. Onshore culture is identified as the optimal method for enhancing seaweed food safety, albeit with a trade-off of reduced seaweed output. Looking ahead, this necessitates cost-effective cultivation technologies, both offshore and onshore, that simultaneously improve food safety and reduce production costs [142,143].

Despite these challenges, there are significant opportunities in the research and development of drugs from seaweeds for the treatment of diabetes, including abundant source of bioactive compounds since seaweeds are a rich source of bioactive compounds, including polysaccharides, peptides, phlorotannins, and pigments, which have been shown to have antidiabetic activity [5,70]. Seaweed-derived bioactive compounds can provide a source of novel drug candidates with different mechanisms of action than the currently available antidiabetic drugs [82,102]. However, there is a need to conduct a SWOT analysis to choose the best compounds based in the above information in order to promote an economically feasible compound which is stable and very well-known and purified.

The seaweed compounds can be a sustainable and environmentally friendly solution in the future. Seaweeds are a sustainable and environmentally friendly source of bioactive compounds compared to traditional medicinal plants [141,144].

So, the research and development of drugs from seaweeds for the treatment of diabetes present both challenges and opportunities. With the identification of specific active compounds, standardization of extracts, and safety evaluation, seaweeds can provide a valuable source of novel antidiabetic drugs [133,145].

## 7. Conclusions and Future Perspectives

Some of the edible seaweeds and seaweed compounds included in this review are simply those that have been studied for nutraceutical reasons. Despite significant effort in generating novel goods, few industrially competent products exploiting these nutraceuticals for human health and well-being have been established. Nutraceutical support for foods needs to further evolve using pharmaceutical techniques and analysis to support and create new safety measures in the food and nutraceutical area to protect humankind from misconceptions.

Diabetes might be treated using drugs that inhibit the enzymes α-glucosidase and α-amylase, which convert starch into glucose before it enters the blood circulation [59]. It is critical to seek effective therapeutic natural drugs that have fewer negative effects. In this case, one seaweed compound (phenolic compound or fucoidan) [59,146] or two conjugated compounds from seaweeds (crude ethanolic extract) [147] can be a key to seaweeds being a natural therapeutic for diabetes. Nonetheless, dieckol increased the activity of antioxidant enzymes in liver tissues, including superoxide dismutase (SOD), catalase (CAT), and glutathione peroxidase (GSH-px), as well as the phosphorylation of AMPK and Akt in muscle tissues, implying that dieckol could be developed as a therapeutic agent for type 2 diabetes [123,128]. Dieckol is the only seaweed compound to be available commercially to treat cardiovascular and obesity diseases [128]. 

However, as demonstrated above, there are other seaweed compounds which can be studied further for diabetes and associated disease amelioration therapeutics. Therefore, more research is needed to investigate the bioaccessibility and bioavailability of these bioactive compounds for long-term positive effects. To assess the bioavailability of nutrients and bioactive substances in seaweed, understanding their content, structure, interactions with other dietary components, and fate in the human body postingestion is crucial. Developing systems that can provide comprehensive data on these aspects is necessary for supporting food agencies in evaluating ingredients and their true potential.

Furthermore, to recover these active compounds from seaweed biomass, cost-effective farming technologies, upstream biomass processing, and greener and environmentally friendly extraction approaches/technologies are required. There is still a long road ahead until seaweed can be a commercial drug exploitation source meeting all safety measures.

## Data Availability

Not applicable.
